# Routine Outcome Monitoring from Psychotherapists’ Perspectives: A Framework Analysis Study of Expected Benefits and Difficulties

**DOI:** 10.1007/s10488-024-01350-w

**Published:** 2024-02-14

**Authors:** Jorge Valdiviezo-Oña, Alejandro Unda-López, Adrián Montesano, Chris Evans, Clara Paz

**Affiliations:** 1 Grupo de Investigación Bienestar, Salud y Sociedad, Universidad de Las Américas, Quito, Ecuador; 2 Departamento de Psicología, Sociología y Trabajo Social, Universitat de Lleida, Lleida, Spain; 3 Faculty of Psychology and Educational Sciences, Universitat Oberta de Catalunya, Barcelona, Spain; 4 School of Psychology, University of Roehampton, London, UK

**Keywords:** Psychotherapists’ expectations, Routine outcome monitoring, Latin America, Framework analysis, Mental health services

## Abstract

Psychotherapists’ attitudes and expectations towards routine outcome monitoring can impact the likelihood of its fruitful implementation. While existing studies have predominantly focused on Europe and North America, research in Latin America remains limited. The aim of this study is to explore therapists’ expected benefits and difficulties prior to implementing a routine outcome monitoring system in a university psychotherapy service in Ecuador. An exploratory and descriptive cross-sectional qualitative study was carried out with 20 participants aged 21 to 47. Semi-structured interviews were conducted to explore participants’ knowledge about routine outcome monitoring, their expectations of the difficulties, benefits, usability and risks of using a monitoring system, as well as their expectations about their role in the process. The data analysis adhered to the framework analysis methodology, leading to the identification of six overarching themes, 36 specific themes and 82 subthemes. The interviews highlighted a spectrum of positive and negative expectations at several levels: institutional/general, therapeutic process, therapists, and clients. Perceived benefits encompassed various aspects, including the availability of supplementary information, continuous adaptation of the therapeutic process, facilitation of organization and treatment planning, opportunities for professional development, and fostering patient trust and empowerment. On the other hand, anticipated difficulties comprised concerns such as discomfort among therapists and clients, increased workload, system usage and technical difficulties, and the risk of excessive quantification. Overall, participants expressed more anticipated benefits than difficulties. We present recommendations that can support and optimize the current local implementation efforts of routine outcome monitoring, both within this service and in other settings. These recommendations consider ways to meet positive expectations and address or mitigate negative ones.

**Supplementary Information:**

The online version contains supplementary material available at 10.1007/s10488-024-01350-w.

## Introduction

Routine Outcome Monitoring (ROM) involves the systematic tracking of patient progress during treatment using standardized measures that focus on evaluating treatment outcomes and can be used to improve them (Howard et al., 1996). The implementation of ROM in real-world settings enables the collection of data to address clinically relevant questions in psychotherapy research, fostering communication between therapists and researchers. Clinicians can utilize the collected data to receive feedback on their practice, to respond relevant treatment-related questions and research findings can be shared with clinicians to maintain an ongoing research-clinical feedback loop that generates evidence supporting the delivery of appropriate mental health interventions. As demonstrated by Låver et al. (2023) in their qualitative meta-analysis of how therapists and patients use self-reported data in psychotherapy, ROM findings can serve various purposes. These include functioning as indicators for assessment, monitoring, and therapy planning; acting as a resource to foster self-awareness, reflection, and influence over patients’ emotional states; serving as a catalyst for interactional processes related to communication, exploration, ownership, alliance, or disruption; and identifying how patients express their purposes, motives, and strategies. For instance, deliberate strategic responses may be employed by clients to achieve specific outcomes, such as ensuring access to services or fulfilling interpersonal desires such as being well-liked.

High-income countries with significant research investment in mental health services have implemented ROM in several settings and for different purposes. However, this is not the case for low-and-middle-income countries (Burgess et al., 2015; Kisely et al., 2015; Roe et al., 2022; Ryan et al., 2020; Smith & Baxendine, 2015; Waldron et al., 2018). In Latin America, a region characterized by economic, cultural and social diversity, some efforts have been made to implement ROM in clinical services (e.g., Dogmanas et al., 2022; Fernández-Alvarez et al., 2015; Gómez et al., 2022; Gómez-Penedo et al., 2023; Valdiviezo-Oña et al., 2022; Zúñiga-Salazar et al., 2021). Nevertheless, there are still limited resources available to expand ROM implementation to more services and settings in the region.

Several previous studies have shown that the likelihood for fruitful ROM implementation depends on the way that organizations approach the process as well as on the attitudes and expectations that therapists have towards it (Mellor-Clark et al., 2016). Some studies have addressed therapists’ and/or patients’ views and attitudes towards ROM systems after or during their implementation (e.g., Solstad et al., 2019), as well as their perceptions and expectations about ROM systems before or at the beginning of their implementation (e.g., Moltu et al., 2018; Norman et al., 2014; Van Wert et al., 2021). In most of these studies both benefits and challenges or obstacles related to the use of ROM or the implementation of ROM systems are explored and reported with varying degrees of acceptability. Some clinicians might experience the use of ROM as an external performance control, as a threatening personal challenge, as unsuitable for patients with complex issues, and as an obstacle that hinders the relationship between patient and therapist (Boswell et al., 2015; Kaiser et al., 2018). ROM has also been perceived as having complexities related to concerns about extra work and time constraints, limitations and administrative burden, depersonalization, ethics, and implementation issues. These complexities may contribute to lower usage of standardized monitoring instruments and result in poorer therapist satisfaction with ROM systems (Ionita et al., 2020; Lutz et al., 2015; Norman et al., 2014; Rye et al., 2019).

Kaiser et al. (2018) found that therapists generally viewed outcome monitoring as useful for providing feedback. However, they perceived process monitoring as too complex, indicating a potential barrier to its implementation. On the one hand, process monitoring involves systematically assessing clients’ progress — often on a daily basis — using psychometrically sound instruments, providing feedback to therapists regarding the client’s state. This approach enables the detection of patterns of change by collecting detailed information on how clients experience and integrate their therapy (Kaiser et al., 2018). On the other hand, outcome monitoring focuses on assessing the effectiveness of the treatment by collecting information, such as symptom severity and the quality of the therapeutic relationship, typically on a weekly basis or before and after treatment (Kaiser et al., 2018; Tasca et al., 2019).

Though challenges vary across specific settings and contexts, certain commonalities exist. Identified common needs include the requirement for adequate support systems, addressing concerns, improving interpretability of monitoring plots and reports, leadership and coordination in the implementation process, utilization of web-based measurement systems and provision of comprehensive training, guidelines, and organizational resources from the outset and throughout the whole implementation process. These measures are crucial for effectively addressing these barriers (Cooper et al., 2021; Gómez-Penedo et al., 2023; Kaiser et al., 2018; Norman et al., 2014).

The identification and understanding of practitioners’ attitudes and expectations towards ROM are pivotal in the implementation process. Understanding these attitudes and expectations helps tailor the system to practitioners’ needs and fosters a positive practitioner-researcher feedback loop. While positive attitudes promote practitioners’ engagement, detecting and addressing misconceptions, misinformation, or biased beliefs is crucial. Addressing both positive and negative expectations within specific cultural contexts and at each stage of introducing ROM is essential to mitigate potential obstacles to uptake (Barkham et al., 2023; Lutz et al., 2022).

In general, studies have mainly been conducted in Europe (e.g., Kaiser et al., 2018; Moltu et al., 2018; Norman et al., 2014; Rye et al., 2019; Waldron et al., 2018) and North America (e.g., Cooper et al., 2021; Ionita et al., 2020). To our knowledge, only a single study has explored and reported therapists’ attitudes towards ROM in Latin America after the implementation of the system (Gómez-Penedo et al., 2023). In that study, therapists perceived the implementation of ROM and feedback as useful, providing a complementary perspective and enabling supervision.

Aiming at expanding ROM implementation in Ecuador and Latin America, we are using a web-based ROM system, developed for collecting and analyzing routine monitoring data (MarBar: https://www.marbarsystem.com/). The system offers written and graphical information on client progress at various levels: for clinicians, services and researchers; it scores and graphs the data, provides a brief written report to aid interpretation and shows a cut-off score that separates participants in two groups: those with scores that are typical of the helpseeking/clinical population, and those with scores that are typical of the non-help-seeking/non-clinical population.

The development of the ROM system we are now using builds upon previous psychotherapy research efforts in Ecuador, which is an emerging field in the country. Paz et al. (2021) conducted a scoping review to identify the outcome and change measures used in Latin America; in doing so, they identified a notable gap as no studies had reported on the use of these measures specifically in Ecuador. Paz et al. (2020a) also explored the perceptions of mental health service users in the country regarding an outcome measure, namely the Clinical Outcomes in Routine Evaluation-Outcome Measure (CORE-OM) and identified that participants found this measure to be comprehensible and valuable for assessing psychological distress and treatment progress. The authors remarked that clinicians should bear in mind that scores are relationally and contextually constructed within the local organizational and cultural framework.

Following their exploration of mental health service users’ views on outcome and change measures, Paz et al. (2020b) examined the psychometric properties of the Spanish version of the Schwartz Outcome Scale-10 (SOS-10-E) and Paz et al. (2020c) those of the CORE-OM, assessing their acceptability, reliability and validity in the country. Subsequently, the CORE-OM was employed for ROM in two psychological centers in Ecuador, investigating changes in clients’ psychological distress (Valdiviezo-Oña et al., 2022; Zúñiga-Salazar et al., 2021).

The present study builds upon these foundations and aims to explore therapists’ expected benefits and difficulties prior to implementing a monitoring system in a university psychotherapy service. We hope to provide insights that can guide the development of effective strategies to overcome barriers and promote the adoption and implementation of ROM of mental health interventions in similar centers, in Ecuador and Latin America.

## Methods

### Design

This is an exploratory and descriptive cross-sectional qualitative study. Data analysis was carried out following the framework analysis methodology (see Analysis section for a detailed description) as it provides a systematic approach to data management and lays out a flexible structure in which data can be interpreted and analyzed. Furthermore, conclusions that can be drawn from the obtained data are influenced by the interpretations of the researchers involved in the process (Gale et al., 2013).

### Context and Setting

This study is part of a larger naturalistic, longitudinal, exploratory, and descriptive project that seeks to systematically examine the progress and outcomes of treatment at the *Centro de Psicología Aplicada* (CPA; Center for Applied Psychology) of the Universidad de Las Américas in Ecuador (Clinical Trials registration: NCT05343741; Valdiviezo-Oña et al., 2023)

A co-therapy model is utilized at the CPA, where trained psychotherapists and psychology trainees collaborate using different psychotherapeutic models such as systemic, cognitive-behavioral, and integrative (for further information, refer to Valdiviezo-Oña et al., 2022). Additionally, there is a team of supervisors who oversee the progress of all clients. This setting presents the advantage of providing varied narratives over the studied topic coming from psychotherapists, trainees and supervisors.

### Participants

Participation was voluntary and sampling was not carried out, as we invited all the therapeutic staff working at the center in October 2022 and all accepted to participate. A total of 20 participants were included in the present study: nine therapists (45%), nine clinical psychology trainees (45%) and two clinical supervisors (10%). Of these, 13 participants were women (65%; five therapists, seven trainees and a clinical supervisor). The participants’ ages ranged from 21 to 47 (*M* = 28.40 [95% CI 25.45, 31.85]; *SD* = 7.37) with a median age of 26. The mean therapeutic experience years ranged from 0 to 15 years, the mean was 3.38 ([95% CI 1.53, 5.48]; *SD* = 4.54), with a median of 2 years.

### Procedure

On September 29th, 2022, we contacted all participants via email, informed them about the study, requested them to fill out a virtual informed consent in a Microsoft Forms form and asked them to provide a mutually convenient time for an individual semi-structured interview. The interviews had predefined, open-ended questions and free follow-up questions to clarify and deepen in the participants’ testimonies (Ayres, 2008). The interviews were designed to last a maximum of 30 min to minimize participant burden. The questions were envisioned to cover several topics, including knowledge and expectations about ROM, therapists and trainees’ expectations about their role in the ROM process, as well as their perceptions of the usability, risks, and benefits of using a ROM system in the service.

The staff members already had access to the ROM system, but no quantitative data had been collected with it at the point of the interviews. The interviews were carried out by two trained interviewers (first and second authors) between October 3rd and October 7th, 2022, via Microsoft Teams. Prior to the interviews, all participants completed an informed consent form. After completing the informed consent process, all interviews were recorded.

We employed various actions seeking to improve the quality of the interviews. We aimed to enhance the credibility of our findings by using open-ended questions. To ensure dependability — the consistency and stability of our qualitative findings — we used interview protocols and ensured that both researchers involved in data collection were trained in the methods. We also documented all decisions and processes related to data collection.

Our approach aimed to maintain a non-judgmental stance during the interviews and ensure that all interviews covered the same topics to promote potential confirmability. Regarding transferability, we believe that by conducting interviews with all therapeutic staff members from the service our findings can be relevant to other training services in the region with similar organizational structures.

### Data Processing and Analysis

Various software tools were used for data management and analysis. Microsoft Teams provided preliminary automatically generated transcripts. These raw transcripts were edited and corrected in a word processor, and the data was anonymized for further analysis.

The framework analysis method, which involves a process of familiarization, identification of a thematic framework, indexing, charting, and mapping/interpretation, as outlined by Gale et al. (2013), was used to analyze the semi-structured interviews conducted in this study. This approach, commonly used in health research and for analysis of semi-structured interview transcripts, allows for several researchers to compare across and within each set of information, while obtaining a comprehensive overview of the entire information (Parkinson et al., 2016).

Various approaches could have been employed to understand the perspectives of therapists and trainees. For instance, Interpretative Phenomenological Analysis (IPA) could have been considered due to its focus on experiences. However, IPA’s idiographic approach is better suited for a smaller and more homogeneous number of interviews. Grounded theory, which aims to explore social processes and develop theories to comprehend them, could have been an option. Nevertheless, it may be less suitable for our specific research question. While it can be valuable for understanding broad processes, it may not provide the depth and richness needed to explore the subjective expectations and meanings associated. Ultimately, we opted for framework analysis because it provided flexibility and allowed for comparison across diverse cases. This choice was made considering the varied nature of the data and the need for adaptability in analysis (Parkinson et al., 2016).

To conduct the framework analysis, the first and second authors read and familiarized with the data. They then labeled five interviews (two clinical psychology trainees, two therapists, and a clinical supervisor) and attempted to identify and note relevant topics addressed by the interviewees to propose an initial set of codes. Subsequently, they discussed the preliminary codes coming from this process, checked the interview guide and reviewed literature on the topic to further refine this set of codes. Based on these efforts, they proposed an initial analytical framework.

Following this, two authors (third and fifth) reviewed the proposed framework and suggested changes and adjustments. Considering those suggestions, an analytical framework consisting of 36 themes and 82 subthemes grouped in six overarching themes was structured. Additionally, we wrote a brief description of the meaning of each theme, along with examples illustrating what could be coded under those themes and subthemes.

The above-mentioned analytical framework was then applied to all transcripts by two trained reviewers who were responsible for coding, charting and analysis (first and second authors). The goal of using this approach was to identify similarities and differences in the coding of the information and focus on the relationships between different parts of the data. This process enabled us to maintain the context of each research participant’s perspective (Gale et al., 2013).

Data coding was conducted using ATLAS.ti Web (v.23; ATLAS.ti Scientific Software Development GmbH, 2022); and data charting was conducted on a spreadsheet. Both authors independently coded the same interviews and compared codes; whenever there were coding discrepancies, these authors discussed to reach consensus. Following the framework analysis method, after coding was done and coding discrepancies were solved, we created a summary matrix containing a summary of all quotes for each theme by participant, with one participant per row and one overarching theme per column. This allowed to inspect the data both within individual cases and across cases. Finally, quotes from the interviews were analyzed and reported to further clarify the findings.

### Researchers’ Characteristics and Reflexivity

In conducting the interviews for this study, the researchers involved in the data analysis were early career researchers (first and second authors), and they were supported in the process by more experienced researchers (third, fourth and fifth authors). All authors actively contribute to research initiatives aimed at implementing ROM across diverse settings and one uses ROM in his therapeutic practice.

Four of the researchers are men, and one is a woman, and there is diversity regarding regional and ethnic origin, with three researchers from Ecuador and two from two different European countries (Spain and United Kingdom). This composition of the research team opens the way for considering several different insights when reporting the findings and discussing their implications.

Reflexivity and self-awareness were fundamental aspects throughout the research process, so researchers tried to be mindful of their individual perspectives and underlying assumptions. This was evident, for example, during the initial phase of familiarizing with the interviews, where authors engaged in in-depth discussions and collaborated iteratively to structure the framework. Furthermore, during the coding process, active reflection was consistently employed, involving the reevaluation of themes assigned to some quotes. This reflection occurred both during independent coding and when addressing disagreements between coders. The more experienced researchers also fostered reflection among the early career researchers, jointly identifying any gaps in the interpretation and reporting of findings and providing feedback to address them.

While reflexivity was encouraged, we explicitly avoid claiming objectivity in presenting our findings. It is essential to acknowledge that all authors have prior involvement in ROM-related research, and most authors maintain a favorable stance on the implementation of ROM. This predisposition might have introduced preconceived notions that could influence the interpretation of the results. However, one of the authors is quite skeptical about embedded change measurement ─ the process of routinely completing outcome measures within therapy and using the scores to drive or change the process of therapy ─ as a generic approach for all sorts of therapy (Evans, 2012; Evans & Carlyle, 2021).

## Results

In total, six overarching themes, 36 themes and 82 subthemes were identified, with 740 coded quotes. A breakdown of the number of themes and subthemes is shown in Table 1 below.

**Table 1:** Number of themes and subthemes for each overarching theme

Overarching theme	Number of themes	Total number of subthemes
*1. Knowledge about ROM*	4	1
*2. Role in the ROM process*	9	6
Positive expectations		
*3. Useful characteristics of a ROM system*	12	5
*4. Benefits related to the use of a ROM system*	4	31
Negative expectations		
*5. Difficulties related to the use of a ROM system*	4	37
*6. Risks*	3	2

Out of the identified subthemes, *Improvement and continuous adaptation of the therapeutic process* (46 quotes) and *Complementary source of information to the session narrative* (38 quotes), both part of the theme *Benefits related to therapists* were noticeably used on more occasions than other subthemes. For the complete display of overarching themes with their respective themes also including subthemes and their frequencies, please refer to Online Resource 1.

**Fig. 1:**
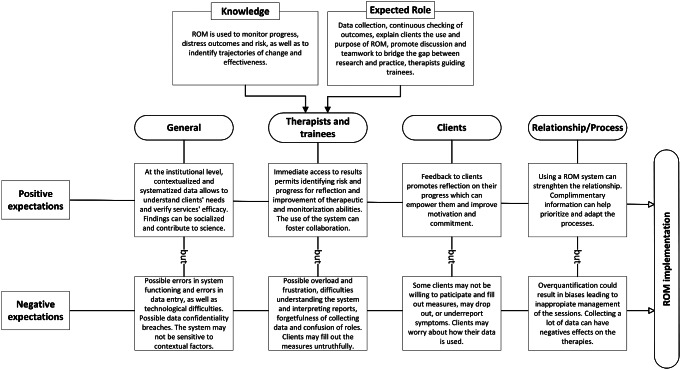
Conceptual model of therapists’ and trainees’ perspectives on ROM implementation

We have developed a conceptual model (see Fig. 1) that summarizes the main findings that emerged from our data analysis. These findings encompass knowledge about ROM; the useful characteristics of a ROM system and the associated benefits (positive expectations), as well as the expected difficulties and risks (negative expectations).

For further detail, below we present specific findings regarding knowledge about ROM, expected role, positive and negative expectations. Participants’ IDs are shown as P and the identifying number (e.g., P12) and overarching themes of positive and negative expectations are in italics.

### Knowledge about ROM

All participants had previous knowledge of ROM with varying levels of knowledge; for example, P13 noted that they did not know about it before arriving to the CPA: “[…] there was none of this at my [previous] internship site. So, it was different, I did not know that monitoring could be done somehow in psychotherapy”.

Most participants reported having previous experience with the use of some form of monitoring in psychotherapy, of whom more than half stated also having experience working with the CORE-OM and CORE-10 as tools to monitor psychological distress levels and identify risk levels.

Regarding possible uses of ROM, almost all participants reported that a ROM system could be used for progress and outcome tracking and one participant asserted that the system could help to identify diversity in the trajectories of change, referring to mental health pathologies as follows:

Mainly for borderline personality disorder and depression. They are the ones who seek help, the ones that will be very emotionally labile and unstable. So, it is a tool that precisely indicates the emotional state they are in (P6).

Whereas some participants see ROM as a mean to identify treatment effectiveness, pointing out that ROM can help them recognize if an intervention was successful and to see immediately the progress in specific indicators such as risk, therapeutic relationship, and explore the impacts related to the use of each therapeutic approach:

[…] knowing what worked, what did not work, what we could improve. Maybe even see what ways of approaching certain problems work better than others, what kind of clients get along better with a specific therapeutic model, what kind of clients work better with a specific therapeutic model (P18).

### Role in the ROM Process

All participants talked about their role in the process, with the most mentioned responsibilities including timely collection of information, ensuring that clients complete the measures usably, and verifying that the monitoring procedures are followed. Nearly half of the participants mentioned the importance of explaining the benefits of ROM and familiarizing clients with ROM’s purpose, with statements like this:

So, maybe at the first moment, to familiarize [them] with — look, these are more or less the questions, this is the reason, it will be useful for this, if you have any doubts when filling it out, please contact me, we can also talk about it during the session —. In other words, that at first the person understands that there is a why and what for and that it is a tool that will not only be useful for me as a therapist, because sometimes it is “well, if it is for you, better ask me during the session”, but it will also be useful for you to realize how you are feeling, how you are progressing through this therapeutic process. (P3)

Some therapists mentioned they expect to act as guides to trainees:

I would help in the sense, maybe to be able to, uh, [to show] how to better manage the information and maybe simplify it a little bit. In the sense, also, of helping the trainees to understand some things better, to help them with terms, to simplify, to make the information much more friendly [for them]. (P16)

Some participants remarked that their role would be to promote discussion in sessions or in supervision, helping bridge the gap between theory and practice. This would entail utilizing the collected evidence to improve the approach to each individual case, continuously reviewing the gathered data, and providing additional information to researchers, if required to contextualize their clients’ outcomes.

### Positive Expectations

Regarding *useful characteristics of a ROM system*, some participants believed that continuous adaptation of the interface of the system is necessary to respond to the variability of each case:

But yeah, I would, I mean, for example, the design maybe some little avatar, something that is much more friendly, that is not so much, that is not an evaluation in itself, but something that can feel like routine, that comes to be considered something that is part of everyday life, as we are used to social networks. Maybe closer to the social network platform that we can just slide and not be like pressing [buttons]. (P13)

A few participants mentioned the importance of also adapting the system to the service and clients’ demands. One participant highlighted the need for regular collaboration between researchers and clinicians to assess the system’s functionalities. In terms of user experience, some participants mentioned that the system should be easy to access and quick to use. Additionally, more than half commented that questionnaires should be easy to fill out and understand, and nearly half of the participants agreed that the monitoring process and the interpretation of results should be easy as well. To summarize this, a participant stated that the system should “be easy to use, that it, that it be understandable, that it be accessible, just, I mean, that it be accessible to all people” (P8).

Also, half of the participants made suggestions regarding variables that should be measured. These suggestions included detailed client clinical information, pharmacological treatment state, therapeutic alliance, a field for clients to express their perspective on their therapy and including assessment of contextual information. They also stated that to be useful the system should focus on monitoring clients’ involvement, distress, and well-being, with some participants addressing how the possibility to compare outcomes across clients could provide a better understanding of the therapy provided:

I consider, for example, that the comparison of psychological cases is very important, not only between my cases but also with those conducted by another therapist. […] Because if I have a case that I feel is not going well and I can compare it with another […], perhaps I could supervise the case with my colleague and understand how I can adapt what he is saying to what I am doing [intervention]. (P5)

Several participants pointed out the importance of having a written report and using graphs with lively formatting for a better understanding of results, such as “colored lines and that it has a description, that is, like what does this mean” (P7). Finally, some participants mentioned that the ROM system could be useful in their private practice “as a tool that complements also the actions of the psychologist” (P5) and one participant believed regular training on the use of the system could be beneficial for its correct use.

Regarding the expected *benefits related to the use of a ROM system*, most participants reported that the use of a ROM system may help them to better adapt to the needs of each client, allowing them to have a more accurate perspective on their cases and make prompt changes. Most participants mentioned as well that the system could provide a complementary source of information to the session narrative “having a process that is also supported by this quantitative part, okay? added to the qualitative part as well” (P10). This additional data was seen as beneficial in enhancing their understanding of client-therapist affinity and in obtaining a more comprehensive view of clients’ progress. It was noted that this information aids in reflecting on effective techniques and tools tailored to specific processes, clients, or symptoms, ultimately contributing to higher quality care. Moreover, participants emphasized that the system could facilitate bridging the perspectives between therapists and clients.

Nearly half of the participants mentioned that they could benefit from data organization and processing within the system. Some expressed a desire for the system to aid in verifying the effectiveness of the service. A few participants specifically mentioned the potential to identify patterns in clients’ progress and characteristics of other therapies. For instance, one participant noted: “the clients reach a certain number of sessions, right?, and then there are very few who continue with the process, so trying to understand what is happening there” (P10), and some reported that the obtained results will help them create hypotheses and lead to conclusions, for example, one participant stated: “ […] I think it is very important to understand perhaps the dropout that exists, like maybe investigating the reasons for dropout” (P18). Moreover, nearly half of the participants mentioned that the system will provide evidence of therapy, allowing for quantifiable validation of psychological techniques used by therapists within sessions stating:

I believe having precise data can give a clear vision of the client’s state. So, I feel it will help a lot with that, and more than anything, like, having a more or less, like, much more tangible reality of how it’s going [the process]. (P17)

Participants highlighted immediate data access as a primary advantage, facilitating the prioritization of intervention aspects for more effective tailoring of interventions. This was voiced by more than half of the participants, with some emphasizing how the system could aid this priorization. Some argued that immediate feedback is a significant benefit, as they will “have the information up to date and at hand, so I feel that the greatest benefit is the feedback of information that you will have about your therapeutic process with your client” (P3). Also, several participants remarked on the importance of continuous risk identification, e.g., risk of self-harm, on the importance of client improvement identification, and on the importance of client deterioration identification. Furthermore, multiple participants suggested that the system promotes reflection and collaboration between therapists and trainees, providing insight into areas where clients may require additional help.

Additionally, nearly half of the participants, mentioned that the ROM system could promote “a continuous training and learning system, which is fundamental […] in [therapeutic] practice” (P12). Some participants mentioned the improvement of monitoring abilities —their ability to track and interpret their clients’ progress— “getting to know new tools” (P8). While more than half of the participants mentioned the potential benefit of enhancing therapeutic abilities.

According to the participants, the use of a ROM system could benefit clients in several ways as well. Several participants discussed how clients could use the system to reflect on their growth. They noted that it could assist clients in identifying areas for improvement, and recognize their progress, giving them a sense of accomplishment and empowerment in their treatment journey. Finally, a few participants stated that the system could strengthen the therapeutic relationship. They believed it could enhance trust in the service by promoting collaboration, personalization, and flexibility in addressing the cases.

Participants argued that the system:

[Allows the client] To gain awareness, because in my experience, many things are being approached during sessions, and later […] in the client’s daily living some changes are not necessarily being visualized or recognized as such. (P3)

Several participants mentioned that the system would facilitate giving feedback to the client on the treatment as it allows therapists to “openly discuss the results” (P6). They believed that this openness could potentially increase clients’ motivation and commitment to the process. In summary, the participants suggested that implementing a ROM system could foster client reflection and feedback, allowing for visible progress leading to an increased sense of trust.

From a wider lens, some participants reported that disseminating the general results could be beneficial. They argued that it could serve as evidence of the processes, add to the understanding of psychological variables within the country’s context, help to better understand the client’s needs and to have a deeper understanding of cases as therapists and trainees can have continuous feedback. A few participants also mentioned how the ROM system could be useful in other contexts such as public health settings and other collaborative and therapeutic settings. Likewise, several participants mentioned that the implementation of a ROM system is much needed in the Ecuadorian context as there is limited information on psychotherapy outcomes stating that a benefit is that:

[…] there will be more contextualized research in Ecuador, […] as much of the literature comes from Spain, México, Colombia, Chile […] Cuba [and] Argentina. […] It opens many things [opportunities] for clinical practice. (P15)

### Negative Expectations

In relation to the expected *difficulties related to the use of a ROM system*, nearly half of the participants mentioned that there could be technological and system difficulties. These concerns included “system glitches” (P4) or errors, mainly the system not working at all, or the links to complete the measures not functioning properly, with statements like the following: “if a link does not work, or something like that, I think it would be a problem because I would not know how to address that, what to do” (P18). One person mentioned possible problems creating a user account and some participants mentioned possible technological access problems, such as access to technology or to a smartphone and the difficulty of some clients not having internet access when therapy is online. Additionally, two participants mentioned that some questionnaires may not be easy to access or inaccessible in the system; and one stated that therapists may not have access to information due to errors in data entry. Participants also mentioned that clients may be unsure of how to fill out the measures or that they might not be easy to fill, and one person stated that the system may not be sensitive to contextual factors, as follows:

It seems to me that some variables should be taken into account because they are things that are activated not only at a personal level but also many times social relations or the context of our country generates or elevates symptomatology. So, if we [therapists] can have something that we can also contribute with respect to this, it seems to me very valuable so that the data are not only raw. (P1)

Regarding difficulties related to the therapeutic process, almost half of the participants mentioned at least one concern. These included therapists potentially becoming overly focused on the numerical results shown in the system, fixating on reducing risks risks and improving scores, which could create bias and divert attention from clients’ actual needs. Participants suggested that excessive quantification might lead to preconceived notions about the client, impacting the approach during session and potentially resulting in inappropriate treatment decisions. For example, one participant expressed: “somehow to see the person as an object, as a, you know, a machine that needs to be corrected, right? And it biases us” (P9). Also, the collection of a huge load of information may have negative effects. One participant mentioned: “I think that even influences the therapeutic alliance, that is, no, it does not favor us very much” (P4).

No participant reported being fearful of the outcomes reported in the ROM system reducing their job stability, that is, that their performance in therapy would be evaluated by the center’s director and supervisors and generate problems for them. However, out of 19 participants who reported possible difficulties, one of the most frequent issues was that therapists and trainees may feel overloaded and frustrated with additional responsibilities. This issue could pose significant challenges, especially if, for instance, utilizing the ROM system places a substantial time burden on them. One trainee said:

We are much more than a number and the functions we perform in a place. In this aspect, the internships, then we have many other things that we also think about. And having a lot of burdens regarding something can lead us to many mistakes because we have our heads in many things, and we can make mistakes. (P20)

This frustration could generate other problems such as therapists and trainees not following procedures, not collecting data or forgetting to schedule appointments as stated by several participants.

Therapists and trainees may also have difficulty in understanding and interpreting the system’s graphs and results and ROM processes, with potential challenges especially for older individuals who may not be technologically savvy. Additionally, one participant noted that ROM may not fit with their therapeutic approach (psychoanalysis), one suggested that a therapist’s perception of a session’s success may differ from what the questionnaires reveal, and a few mentioned the possibility of confusion over roles and responsibilities.

Most participants reported possible difficulties related to clients when using a ROM system. Some participants mentioned that clients may underreport their symptoms because of a perceived pressure to provide socially acceptable responses. However, the most common concern mentioned by half of the participants was that some clients may stop filling out the measures altogether or not fill them at all from the beginning. Forgetfulness was identified as one cause mentioned by one participant, while several participants highlighted reasons such as lack of interest or motivation, as well as discomfort and fatigue associated with the ROM process. For example, a participant said:

The truth is, the main complication that I could think of is not so much the management of us as therapists, trainees, as CPA, but actually getting some clients to fill out the information, because they do not want to, I mean, — no, you know, I just forgot —, — no, you know, I was super busy, but I will, I will get to it later—. (P3)

Of course, incomplete data would result from this. Two participants also mentioned that clients may not recognize benefits related to using the system, leading to reduced willingness to participate or reduced commitment to the therapy. Consequently, this could lead to some clients opting out of treatment.

More than half of the participants reported potential *risks related to the use of a ROM system*. The primary concern raised was regarding data confidentiality, with a few participants mentioning the risks of data breaches, hacking, and non-informed use of the data:

[…] data confidentiality. The fact that these, the results obtained remain confidential that, I do not know, that is, that these results are not used for anything else, for any purpose other than the research that is informed to the clients. (P6)

Some participants also reported that clients may fill out the questionnaires carelessly. Two participants reported possible errors in data entry by therapists or trainees in charge. Furthermore, one participant mentioned that some therapists may fill out questionnaires for clients. All these situations could lead to inaccurate results and introduce a bias into how therapists approach each case.

## Discussion

This study aimed to explore the expected benefits and difficulties of therapists and trainees at the outset of the implementation of a ROM system in a university psychotherapy service in Ecuador, using a qualitative approach to gather detailed insights from participants. This research study represents a novel and relevant endeavor in both the country and the region, contributing to understanding therapists’ and trainees’ perspectives at the initiation of implementation and utilization of a ROM system. Importantly, this study delves beyond the scope of individual outcome measures, focusing on a broader system of data collection. Hence, this paper adds to the sparse literature on therapists’ perceptions towards ROM in Latin America. Notably it stands as the second paper from the region on the topic and the first one to address expectations.

Our findings are mostly in line with previous studies conducted in Europe and North America. For instance, participants pointed out similar perspectives to previous studies (e.g., Ionita et al., 2020; Kaiser et al., 2018; Solstad et al., 2019) regarding the potential improvements in their work and the benefits clients could derive from ROM implementation. These benefits predominantly involve providing additional information, fostering client involvement and structure, and facilitating open discussion. However, we observed some discrepancies from prior studies. Unlike the findings of Kaiser et al. (2018), our results did not indicate that therapists or trainees perceive the implementation of this system as a means of performance control, rather, some participants in our study emphasized the importance of ROM implementation in Ecuador, given the scarcity of psychotherapy outcome studies in the country. Participants perceived that ROM implementation creates opportunities that could foster scientific advancement within the country, a noteworthy contrast with studies conducted in Europe and North America where these aspects are not commonly addressed by participants. This disparity may be attributed to the emerging nature of psychotherapy research in Latin America, coupled with economic inequalities across regions, evidenced for example in lower national research investment and limited access to research funding for studies (Ciocca & Delgado, 2017). In contrast, North America and Europe exhibit substantially higher levels of scientific and technological development, along with increased funding for psychotherapy research. Besides that, no specific differences regarding the implementation of a ROM system that could be attributed to cultural factors were identified in the collected data. In-depth analysis of the results is presented in the following sections.

### Previous Knowledge and Expected Role

Participants stated they all had previous knowledge about ROM for monitoring psychotherapeutic outcomes, treatment effectiveness, or trajectories of change. Most participants were familiar with CORE measures and all participants talked about their role in the process, which for them, mainly would involve collaborating in the collection of timely and accurate information, ensuring clients correctly fill out the measures, and verifying adherence to the necessary monitoring procedures. The center’s staff also reflected on how the use of ROM tools could aid in discerning how a particular treatment could influence psychotherapeutic outcomes and help identify learning opportunities. Previous studies have deemed training and previous knowledge as some of the main facilitators for the implementation of similar systems, specifically understanding and familiarity with procedures and ROM tools’ (Ionita et al., 2020; Kaiser et al., 2018). This existing knowledge base among participants could provide an advantage in the current endeavor to implement a ROM system. Given participants’ previous familiarity with ROM and experience using outcome measures, it could potentially expedite training and comprehension of the system.

### Positive Expectations

The therapists and trainees included in our study considered one of the main potential advantages of using a ROM system that it can serve as a supplementary source of information that helps them to reflect on their strengths and weaknesses and as a learning resource to improve their skills and obtain information about clients prior to sessions. Additionally, they emphasized its role in enhancing visibility regarding changes throughout therapy, supporting clinical decision-making, facilitating organization and treatment planning, and fostering trust and openness between clients and therapists. These findings align with existing literature (Kaiser et al., 2018; Moltu et al., 2018; Norman et al., 2014; Rye et al., 2019; Sharples et al., 2017).

The expectations reported in our study are also congruent with previous studies which have suggested that ROM is useful for: (1) providing feedback with relevant information for process evaluation and adaptation; (2) assessing specific treatments and approaches used in a particular setting; (3) comparing success rates; (4) providing a sense of accountability; (5) promote focus and involvement in work, (6) offer a comprehensive perspective on success rates; and (7) assess if clients’ needs and goals have been met (Gómez-Penedo et al., 2023; Norman et al., 2014; Sharples et al., 2017).

In the present study, the ROM system was perceived as a tool that will provide real-time feedback not only to therapists but also to clients on their progress and outcomes. Clients who receive feedback may experience increased awareness of symptoms, goal attainment, self-efficacy, and communication with their therapist, leading to a more active and empowered involvement in the process (Cooper et al., 2021; Kaiser et al., 2018).

Consistent with our findings, an analysis of qualitative studies around the implementation of ROM and similar systems conducted by Solstad et al. (2019), shows that the opportunity to engage clients by empowering them, as they participate in setting goals and evaluating their own progress are among the main expected outcomes. Additionally, Solstad et al. (2019) mention that the use of ROM systems could improve collaborative practices, promoting open discussion for both clients and practitioners by providing structure, focus and prioritization of clients’ needs.

### Negative Expectations

Even though there may be several benefits associated with ROM, the implementation of ROM systems can also present some challenges and barriers both on the side of therapists and of clients. Specifically, our study revealed that most participants anticipated potential challenges such as feeling overwhelmed and discouraged by additional responsibilities, consistent with previous literature that have reported perceptions of increased workload involving questionnaire administration, results analysis and extended working hours (Gleacher et al., 2016; Ionita et al., 2020; Kaiser et al., 2018; Norman et al., 2014).

Regarding clients, other studies have indicated that they may have difficulties related to ROM implementation as well. Clients may feel stressed by completing questionnaires, by frequent assessment; there may be negative impacts on the therapeutic alliance and some of them may feel pressured to provide positive responses to the measures arising from clients’ perceived necessity to create a false impression of well-being, driven by concerns of insurance companies discontinuing payment for their treatment (Ionita et al., 2020; Kaiser et al., 2018; Rye et al., 2019). This is congruent with our findings, with therapists stating that clients may act on that pressure wanting to avoid drastic clinical actions such as hospitalization or referral. It is relevant to note that this aspect differs significantly between high-income and lower income countries, with many of the former having universal or nearly universal public health coverage and private insurance companies covering psychological treatments. By contrast, in lower income countries like Ecuador, neither the public health system nor private insurance companies fully cover psychological care to begin with (Bayarsaikhan et al., 2022).

Another common concern that emerged in our study was related to system usage and potential technical difficulties. These concerns are also echoed in the existing literature regarding therapists’ attitudes towards ROM, with issues such as: complex designs of the systems, difficult-to-comprehend language, challenging management and interpretation of instruments and reports (Gleacher et al., 2016; Kaiser et al., 2018; Sharples et al., 2017).

Therapists and trainees in our study were also concerned about the risk of over-quantification, that is, reducing clients’ progress and state to the quantitative results of questionnaires’ results. This finding aligns with previous research suggesting that monitoring may not fully account for various relevant, complex and influential factors, or be used as a replacement for clinical judgement (Ionita et al., 2020; Kaiser et al., 2018; Norman et al., 2014). This is especially relevant, considering that political, social, and economic factors can significantly impact the mental health of individuals and communities (Moncrieff, 2022). If the focus of outcome monitoring is on individual symptoms without considering the broader social and political context in which these symptoms arise, it can lead to a limited understanding of clients’ issues and their underlying causes.

Finally, previous studies have analyzed mental health professionals’ concerns regarding ROM, finding that its implementation could be perceived as micromanagement, and as a means of control or therapy performance, not intended with a clinical or therapeutic benefit in mind, thereby decreasing the probability of continuous use of the system (Goldberg et al., 2016; Kaiser et al., 2018). Nevertheless, this is not consistent with our findings as no participant perceived the incoming system implementation in such way, which may have to do with research culture and measurement as a routine practice in psychotherapy in Ecuador still being in an incipient exploratory state.

Overall, we found that therapists identified more potential benefits than difficulties and risks coming with the implementation of a ROM system. Also, they mentioned that a proper framing regarding the aims of ROM and its use within the center could generate adherence, and that having continuous training on the use of the system, data interpretation and receiving continuous feedback could maximize the attained benefits. As this study is part of a larger naturalistic project, its findings help to reflect on how to adapt the ROM system and data collection procedures, considering participants’ needs, by harvesting existing resources and helping to identify possible weaknesses to be addressed.

### Implications for Future Research and Practice

Based on the positive and negative expectations described by participants in our study and informed by previous research, we present below several general recommendations that may facilitate future ROM implementation. First, we recommend clearly communicating the purpose, objectives, and potential benefits, disadvantages and risks of ROM to the professionals involved (Kaiser et al., 2018), considering that professionals who have a positive attitude towards ROM are more likely to maintain its use (Waldron et al., 2018). Second, we suggest fostering continuous training and capacity building for clinicians who administer ROM instruments, as this can increase their level of self-efficacy and involvement (Ionita et al., 2020; Kaiser et al., 2018; Rye et al., 2019; Sharples et al., 2017). Training courses and follow-up sessions are recognized as important facilitators for the implementation of ROM to minimize aversion (Rye et al., 2019). Third, we propose providing organizational support and incentives for the use of ROM, such as educational credits or recognition (Rye et al., 2019). We also recommend enhancing communication and collaboration within therapy, as this can improve the integration of ROM into clinical practice (Solstad et al., 2021).

Moreover, it is crucial to have a user-friendly system that requires minimal time to use and complete the measures. Gómez-Penedo et al. (2023) and Ionita et al. (2020) argue that this can reduce the burden on both clients and therapists, promoting continuous use of the system. Another way to enhance the usefulness of ROM is to provide comprehensive reports, both for therapists and for clients, including information about clients’ well-being and functioning beyond clinical symptoms or diagnosis. Moltu et al. (2018) suggests that measuring early signs of progress or change can benefit both clients and therapists; for instance, indicators related to risk and symptom monitoring can help track progress and inform treatment adjustment. In line with this, the software we are using to collect the data portrays clients’ progress through graphs and provides a brief written report. This software does not include expected trajectories of recovery (ETRs), however, the larger project of which this study is a part represents an initial phase towards establishing a comprehensive database that may allow to develop normative trajectories of change and ETRs. Future inclusion of ETRs in the system could provide guidelines to clinicians for interpreting the data (Valdiviezo-Oña et al., 2023).

In a broader sense, we also recognize a need to conduct more ROM-related research in Ecuador and the region. Latin America is very diverse both between and within countries, hence contextualized research on the subject that considers specific strengths and limitations is needed to conduct comparative studies that examine the cultural and contextual factors influencing the implementation of ROM. Several efforts such as establishing regional networks or consortiums that facilitate collaboration and knowledge exchange among mental health professionals and researchers, joint conferences, workshops, and training programs that promote cross-country learning and sharing of experiences related to ROM could be helpful to identify and address cross-cultural differences.

Another important issue in the region is a lack of or limited funding and/or government support (Becerra-Posada et al., 2021). Therefore, future studies could evaluate how international collaboration including financial and other types of support coming from countries with more history of ROM, such as providing technical assistance, training materials, best practices and mentorship programs could influence ROM implementation and capacity building in countries at the early stages of ROM implementation.

Finally, we provide several specific recommendations (See Table 2) for implementation and tailoring of ROM at the local (CPA) level and in other similar services, considering how to meet the positive expectations and how to mitigate or address the negative expectations.

**Table 2:** Recommendations for ROM systems implementation locally and in other similar services

Level	Recommendations
Local (CPA)	*Comprehensive Training:* • Provide workshops and seminars that focus on the theoretical background and practical application of ROM in psychotherapy. • Use case examples and role-plays to illustrate how ROM can be utilized for different needs and aims. • Offer ongoing training sessions to address any emerging challenges and ensure therapists feel confident in using the ROM system.
	*Iterative Implementation:* • Integrate ROM into therapists’ and trainees practice progressively. • Regularly assess therapists’ and trainees’ experiences and gather feedback on the strengths and weaknesses of the ROM system and data collection process. • Use this feedback to iteratively improve the ROM system, addressing any technical or usability issues.
	*Supportive Environment:* • Foster a collaborative and supportive culture within the service that encourages open dialogue about the benefits and challenges of ROM. • Establish peer supervision or consultation groups where therapists can share their experiences and learn from each other’s successes and struggles.
	*ROM and Feedback Integration:* • Develop clear protocols for incorporating ROM and feedback into therapy sessions and treatment planning. • Encourage therapists to engage clients in discussions about their ROM data, helping them understand the purpose and benefits of the process. • Emphasize the potential for ROM to enhance outcomes and promote client engagement in their own treatment process.
	*Continuous Evaluation:* • Establish regular evaluation processes to monitor the impact of ROM on therapists’ practice and clients’ outcomes. • Use qualitative and quantitative measures to assess therapists’ satisfaction, confidence, and perceived value of ROM. • Adjust the implementation strategy based on the evaluation results to ensure ongoing improvements and address any implementation challenges.
	*Integration of Clinical Judgment:* • Emphasize the importance of integrating ROM data with therapists’ clinical judgment and expertise. • Provide training on how to interpret ROM results in the context of each individual client, considering the complexity of their presenting issues and the broader social and political factors impacting their mental health.
Other similar services	*Assess Organizational Readiness:* • Conduct an internal assessment of the service’s readiness for implementing a ROM system. Evaluate factors such as staff readiness, technological infrastructure, and availability of resources. • Ensure that the service has a supportive culture that values continuous learning and improvement.
	*Select a ROM System:* • Research and select a ROM system that aligns with the service’s specific needs. • Consider factors such as ease of use, compatibility with existing systems, data security, and flexibility in including measures to fit the service’s population and goals. • Seek input from therapists and other stakeholders during the selection.
	*Develop a Clear Implementation Plan:* • Create a detailed plan that outlines the steps, timelines, and responsibilities involved in implementing the ROM system. • Clearly communicate the purpose and expectations of ROM use to therapists and staff members. Training • Familiarize therapists with the technical aspects of ROM implementation, including selecting appropriate outcome measures and integrating them into practice. • Train therapists on how to administer questionnaires. • Address common concerns and challenges related to ROM implementation, such as workload management, client engagement, and interpretation of results. • Provide training on data analysis techniques specific to ROM, such as identifying reliable change, and interpreting progress trajectories. • Teach therapists how to integrate ROM data with clinical judgment and contextual factors to inform treatment decisions and interventions. • Emphasize the importance of considering individual client characteristics as well as broader social and cultural factors when interpreting ROM results.
	*Data Management and Analysis Processes:* • Develop protocols for securely collecting, storing, and managing ROM data, ensuring compliance with data protection regulations and ethical guidelines. • Consider using data visualization tools or dashboards to facilitate the interpretation and communication of ROM results to therapists and clients.
	*Monitor and Evaluate the Implementation:* • Continuously monitor the implementation process to identify challenges, barriers, and areas for improvement. • Regularly gather feedback from therapists and clients to assess their experiences with the ROM system and make necessary adjustments. • Evaluate the impact of ROM on therapy outcomes, client satisfaction, and therapist professional development to assess the effectiveness of the implementation and justify ongoing resource allocation.

### Limitations

Our findings provide insights and guidance by portraying therapists’ specific needs and by tackling possible risks and obstacles that may arise with the implementation of a ROM system. Nevertheless, there are several limitations that need to be considered. The specific cultural and contextual factors in Ecuador may influence participants’ perspectives about ROM implementation and its influence on psychotherapy, meaning results may be transferable only to similar cultural and regional contexts. While we recognize this limitation, we emphasize the usefulness of our findings as these results can prove valuable for the implementation of ROM in mental health services across Ecuador, and given the limited information and experience on ROM implementation within Latin America, our study provides information for future endeavors in the region to adapt their services to accommodate for their specific contextual needs.

Despite efforts to ensure accuracy, there is a risk of translation bias and loss of nuance because we used translated quotes from interviews originally conducted in Spanish. Subtle cultural and linguistic nuances may have been unintentionally altered, affecting the accuracy of the quotes. Translation involves subjectivity and interpretation, making it difficult to capture the original intent and context of the interviews. Caution should be exercised when interpreting these translated quotes.

Another inherent limitation lies in most authors’ pre-existing positive stances towards ROM, coupled with our prior engagement in ROM-related research endeavors. This predisposition introduces a potential source of bias, as our subjective experience could shape the interpretation of the findings. We do not seek to impose our interpretations as truths, but instead, aim to promote multiple nuanced interpretations of therapists’ expectations regarding the implementation of ROM.

Furthermore, our study focuses on exploring the perspectives of therapists and trainees regarding the potential benefits and difficulties of ROM implementation cross-sectionally. While this provides valuable insights into their expectations, it does not capture their perceptions over time (i.e., their experiences with the system, which could be addressed with a follow-up) nor does it encompass clients’ perspectives. Incorporating clients’ views could offer a more comprehensive understanding of the potential benefits and challenges of ROM implementation in the country and the region. In the future, clients’ and therapists’ perspectives on their experience using a ROM system could be explored by interviewing them or asking them to complete surveys.

## Conclusion

The body of research exploring therapists’ expectations prior to the implementation of ROM and/or feedback procedures and systems is notably limited. Most existing studies have predominantly centered on post-implementation explorations, delving into therapists’ attitudes and experiences following the adoption of such systems. Consequently, in light of the scarce research conducted in this domain in general, and particularly within the context of Latin America, this study has the potential of elucidating therapists’ pre-implementation perspectives and offering insights into the expected benefits and challenges associated with the integration of ROM systems in mental healthcare settings that can guide the development of effective strategies to overcome barriers and promote thriving implementation of ROM in psychotherapy in Ecuador and the region.

## Electronic Supplementary Material

Below is the link to the electronic supplementary material.

Supplementary Material 1

## Data Availability

In accordance with the guidelines of the research ethics committee that granted approval for this study and as stated in the protocol study of the project of which this article is a part of, individuals seeking access to the data used in this article must submit a written request to the corresponding author. Suitably qualified researchers may obtain a de-identified dataset in encrypted form. To have access to the data, researchers must (a) obtain ethical approval for their proposed analysis, (b) pre-register their statistical analysis plan, and (c) sign a data sharing agreement that permits data storage and analysis for a limited period.

## Declarations

### Guideline Followed

The Standards for Reporting Qualitative Research (SRQR) guideline was followed in this article.
