# Supplementary material for: Routine Outcome Monitoring from Psychotherapists’ Perspectives: A Framework Analysis Study of Expected Benefits and Difficulties
Source: Adm Policy Ment Health. 2024 Feb 14;52(1):194–209. doi: 10.1007/s10488-024-01350-w (PMC11703935; doi:10.1007/s10488-024-01350-w)
Supplement: Supplementary file 1 — Supplementary Material 1 [file 10488_2024_1350_MOESM1_ESM.docx]

**Supplementary Material**

**Article Title**

Routine Outcome Monitoring from Psychotherapists' Perspectives: a Framework Analysis Study of Expected Benefits and Difficulties

**Journal Name**

Administration and Policy in Mental Health and Mental Health Services Research

**Authors Names**

Jorge Valdiviezo-Oña*, Alejandro Unda-López, Adrián Montesano, Chris Evans, Clara Paz

***Affiliations of the Corresponding Author**

1. Grupo de Investigación Bienestar, Salud y Sociedad, Universidad de Las Américas, Ecuador
2. Departamento de Psicología, Sociología y Trabajo Social, Universitat de Lleida, Spain

**E-mail Address of the Corresponding Author**

[jorge.valdiviezo.ona@udla.edu.ec](mailto:jorge.valdiviezo.ona@udla.edu.ec)

**Online Resource 1**

*Quotes’ Frequencies Divided by Overarching Themes, Themes and Subthemes*

| Overarching theme, themes and subthemes | Quote Frequency | *n* |
| --- | --- | --- |
| Knowledge about ROM | 60 | 20 |
| - Progress follow-up and therapeutic processes outcomes | 26 | 18 |
| - Identification of diversity of change processes | 1 | 1 |
| - Means to measure the effectiveness of treatments | 7 | 7 |
| - Previous experience in the use of monitoring systems | 26 | 15 |
| - - Measures they have used | 18 | 12 |
| Role in the ROM process | 117 | 20 |
| - Supervision and verification of process compliance | 14 | 10 |
| - Continuous review of collected data | 12 | 11 |
| - Discussion of results | 10 | 8 |
| - Communication between therapist and trainee | 10 | 6 |
| - Communication with the client | 32 | 11 |
| - - Explanation of the importance and purpose of ROM | 21 | 10 |
| - - Patient accompaniment and support | 2 | 2 |
| - Communication with supervisors | 3 | 1 |
| - Lack of knowledge of roles in the process | 0 | 0 |
| - Conduct data collection | 9 | 7 |
| - Importance of the role | 27 | 12 |
| - - Promoting the dialogue between evidence and practice | 8 | 5 |
| - - Support and guidance from therapists to trainees | 6 | 5 |
| - - Facilitating data collection | 7 | 6 |
| - - Promoting teamwork | 6 | 3 |
| Positive Expectations |  |  |
| Useful characteristics of a ROM system | 124 | 20 |
| - Continuous adaptation of the system interface | 8 | 4 |
| - Short time spent using the system | 8 | 6 |
| - Variables included in the system | 17 | 10 |
| - Adaptation of the evaluation to the service and the reasons for consultation | 5 | 3 |
| - Risk identification | 9 | 7 |
| - Format for presentation of results | 37 | 18 |
| - - Graph visualization | 7 | 7 |
| - - Ease of monitorization process tracking | 9 | 8 |
| - - Ease of interpretation of reports | 11 | 9 |
| - - Brief summary of results | 3 | 3 |
| - Inclusion of clinical record | 1 | 1 |
| - Implementation of the system in other locations | 4 | 4 |
| - - Use of the system in own clinical practice | 4 | 4 |
| - Monitoring of change | 3 | 3 |
| - Ease of log-in/system access | 8 | 5 |
| - Ease of understanding and answering questionnaires | 17 | 12 |
| - Comparison of psychological cases/processes | 7 | 5 |
| Benefits related to the use of a ROM system | 276 |  |
| - Institutional and general benefits | 43 | 16 |
| - - Data systematization | 8 | 8 |
| - - Socialization of results | 5 | 4 |
| - - Expansion of the use of monitorization in other services | 4 | 3 |
| - - Contribution to psychology scientific progress | 13 | 7 |
| - - Verification of service effectiveness | 6 | 5 |
| - - Identification of patterns of change | 3 | 3 |
| - - Generate hypotheses and conclusions | 4 | 3 |
| - Benefits related to the therapeutic process | 114 | 20 |
| - - Clients’ trust in therapy | 4 | 3 |
| - - Therapeutic objectives monitorization | 1 | 1 |
| - - Priorization of aspects to be addressed | 7 | 4 |
| - - Continuous improvement and adaptation of therapeutic processes | 46 | 17 |
| - - Strengthening of the therapeutic relationship | 3 | 3 |
| - - Source of information complementary to the session narrative | 38 | 15 |
| - - Evidence of therapeutic processes | 15 | 9 |
| - Benefits related to therapists | 98 | 19 |
| - - Feedback for therapist | 5 | 4 |
| - - Continuous learning | 31 | 12 |
| - - - Learning monitoring skills | 6 | 4 |
| - - - Improvement of therapeutic skills | 15 | 11 |
| - - Identifying clients' evolution/progress | 22 | 16 |
| - - - Identification of improvement | 4 | 3 |
| - - - Identification of deterioration | 3 | 3 |
| - - Immediate access to data | 13 | 11 |
| - - Reflection on practice | 16 | 7 |
| - - Understanding clients’ needs | 8 | 6 |
| - - Resource for pre-session preparation | 3 | 3 |
| - Benefits related to clients | 21 | 13 |
| - - Client identifies evolution/progress | 7 | 6 |
| - - Motivation of clients with the therapeutic process | 2 | 2 |
| - - Feedback with the client | 6 | 6 |
| - - Commitment of clients to the therapeutic process | 2 | 1 |
| - - Promotes reflection of clients | 4 | 4 |
| - - Recognition of the quality of care | 0 | 0 |
| Negative expectations |  |  |
| Difficulties related to the use of a ROM system | 151 |  |
| - Technological and system difficulties | 18 | 9 |
| - - Access to internet | 3 | 3 |
| - - Access to the system | 2 | 2 |
| - - - Access to the information | 1 | 1 |
| - - System errors | 7 | 5 |
| - - Limited information on contextual factors | 2 | 1 |
| - - Not having technological devices | 4 | 3 |
| - Difficulties related to the therapeutic process | 20 | 8 |
| - - ROM does not capture the complexity of therapeutic processess | 1 | 1 |
| - - ROM is apt only for individual therapeutic processes | 0 | 0 |
| - - Excessive focus on quantitative outcomes | 7 | 5 |
| - - Negative influence of ROM on therapy/therapeutic relationship | 4 | 2 |
| - - Conditioning of therapy to ROM outcomes | 8 | 5 |
| - Difficulties related to therapists and trainees | 68 | 19 |
| - - Technological abilities | 4 | 4 |
| - - Duration of entry and system use | 4 | 4 |
| - - Lack of knowledge of the use of the platform | 3 | 3 |
| - - Lack of knowledge of the data collection process | 4 | 4 |
| - - Lack of knowledge of the questionnaires | 1 | 1 |
| - - Difficulty in the interpretation of reports | 12 | 7 |
| - - Concern about additional working hours | 1 | 1 |
| - - Therapist overload | 14 | 6 |
| - - - Therapist frustration | 9 | 6 |
| - - Therapists not complying with the procedures | 12 | 7 |
| - - Concern that clients may not answer honestly | 6 | 4 |
| - - Inconsistency between therapist's perception and results in questionnaires | 3 | 3 |
| - - ROM does not fit in with my therapeutic approach | 1 | 1 |
| - - ROM is not necessary | 0 | 0 |
| - - ROM is not useful | 0 | 0 |
| - - ROM is a waste of time | 0 | 0 |
| - - Lack of knowledge/confusion of roles | 3 | 3 |
| - Difficulties related to clients | 55 | 16 |
| - - Perception of lack of usefulness of ROM | 0 | 0 |
| - - Frustration with ROM | 14 | 8 |
| - - Lack of motivation and compliance | 29 | 12 |
| - - - Non-response | 15 | 10 |
| - - - - Missing data | 6 | 5 |
| - - Drop-out from the therapeutic process | 5 | 4 |
| - - ROM is inappropriate/incompatible for some clients | 0 | 0 |
| - - Clients may feel pressured to provide positive responses | 7 | 5 |
| Risks | 20 | 11 |
| - Data confidentiality | 9 | 7 |
| - - Data leaking | 3 | 3 |
| - Validity of data collected | 10 | 6 |
| - - Incorrect reporting of responses | 5 | 4 |
| - Loss of information collected | 1 | 1 |
